# Rosuvastatin Use Implicated in the Drug Reaction with Eosinophilia and Systemic Symptoms

**DOI:** 10.7759/cureus.7098

**Published:** 2020-02-25

**Authors:** Syed Farrukh Mustafa, Meer R Zafar, Timothy W Miller

**Affiliations:** 1 Internal Medicine, William Beaumont Hospital, Royal Oak, USA; 2 Internal Medicine, Sisters of Charity Hospital, Buffalo, USA; 3 Internal Medicine, Jacobs School of Medicine and Biomedical Sciences, Buffalo, USA

**Keywords:** drug reaction, eosinophilia, rosuvastatin, generalized rash, generalized lymphadenopathy, glucocorticoids

## Abstract

Drug reaction with eosinophilia and systemic symptoms (DRESS) is a life-threatening drug-induced hypersensitivity reaction that is most closely associated with antiepileptics and antibiotics. While cases of DRESS are rare, here we present a case of DRESS in an adult male following administration of rosuvastatin who presented with fevers, generalized rash, and facial fullness. Vitals on presentation were temperature 102^o^F, pulse 95/min, blood pressure 95/47 mmHg, and respiratory rate of 14/min. His physical examination revealed scleral icterus, generalized blanching maculopapular rash, facial fullness, and right upper quadrant tenderness. Laboratory investigations found hemoglobin 10 gm/dl, white blood cell count 16.0 K/uL, peripheral eosinophil count 1,700 K/uL, alkaline phosphatase 2,501 U/L, aspartate transaminase 620 U/L, alanine transaminase 680 U/L, total bilirubin 13.2 mg/dl with a direct component of 9 mg/dl, blood urea nitrogen 66 mg/dl, creatinine 5.20 mg/dl, glomerular filtration rate 8 ml/min, and immunoglobulin E level 623 IU/mL. Serology for viral hepatitis, Epstein-Barr virus, cytomegalovirus, and human herpesvirus 6 was negative. Computed tomographic scan of chest, abdomen, and pelvis showed generalized lymphadenopathy. Over the next week, the patient deteriorated clinically with worsening transaminitis and oliguric acute renal failure requiring renal replacement therapy. As per the European Registry of Severe Cutaneous Adverse Reaction Criteria (RegiSCAR), the probability of rosuvastatin-induced DRESS syndrome was scored as “definite.” He was treated with systemic and topical glucocorticoids leading to a gradual improvement in his symptoms. Skin biopsy was suggestive of DRESS syndrome as well. Since DRESS carries such a significant risk of mortality between 10% and 20%, DRESS must be recognized and treated as soon as symptoms present. Clinicians should also be aware that statins, one of the most commonly prescribed drugs, are also a potential cause DRESS.

## Introduction

Drug reaction with eosinophilia and systemic symptoms (DRESS) is a severe, potentially life-threatening drug-induced reaction characterized by fever, skin eruption, hematologic abnormalities, and multi-organ dysfunction [1]. The skin rash can range from a pruritic maculopapular eruption that progresses to a confluent erythematous rash with follicular accentuation, frequently involving more than 50% of the body surface area [2]. Hematologic abnormalities in DRESS include elevated leukocyte count with significant eosinophilia and atypical lymphocytosis [1]. Multiple internal organs can be involved with liver dysfunction being the most common, ranging from transient mild elevation in liver function tests in most cases to fulminant liver failure [3]. Other common manifestations include lung involvement (pneumonitis, pleural effusions) and kidney involvement (interstitial nephritis). According to a study, the estimated annual incidence of DRESS is 0.9/100,000 [4]. It is associated with anywhere between 1/1000 and 1/10,000 drug exposures and mortality is 10% to 20% [5]. Dibek Misirlioglu et al. reported that antibiotics were the most common (50%) medication in the etiology; 87.5% of the suspected antibiotics were beta-lactams and 12.5% were macrolides. Antiepileptics were the second (37.5%) most common class of drugs in the etiology [6]. Statin use has not been commonly associated with DRESS. We describe a case of DRESS in a patient with exposure to rosuvastatin.

## Case presentation

**Figure 1:**
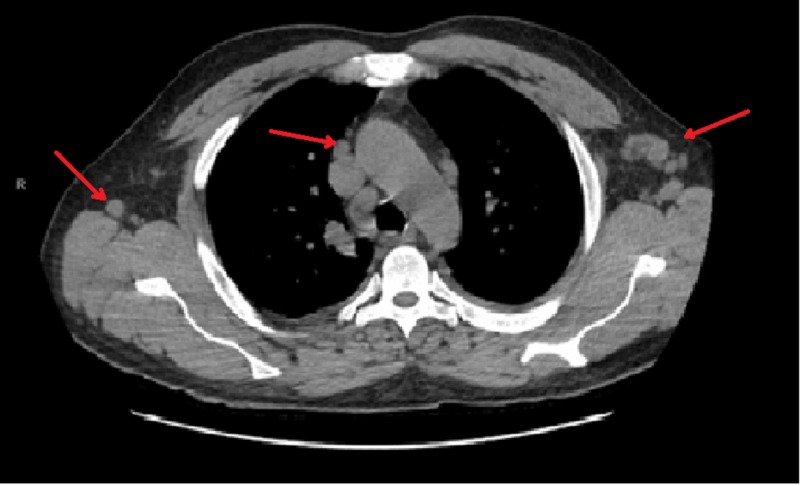
CT scan of the chest showing enlarged mediastinal and axillary lymph nodes.

**Figure 2:**
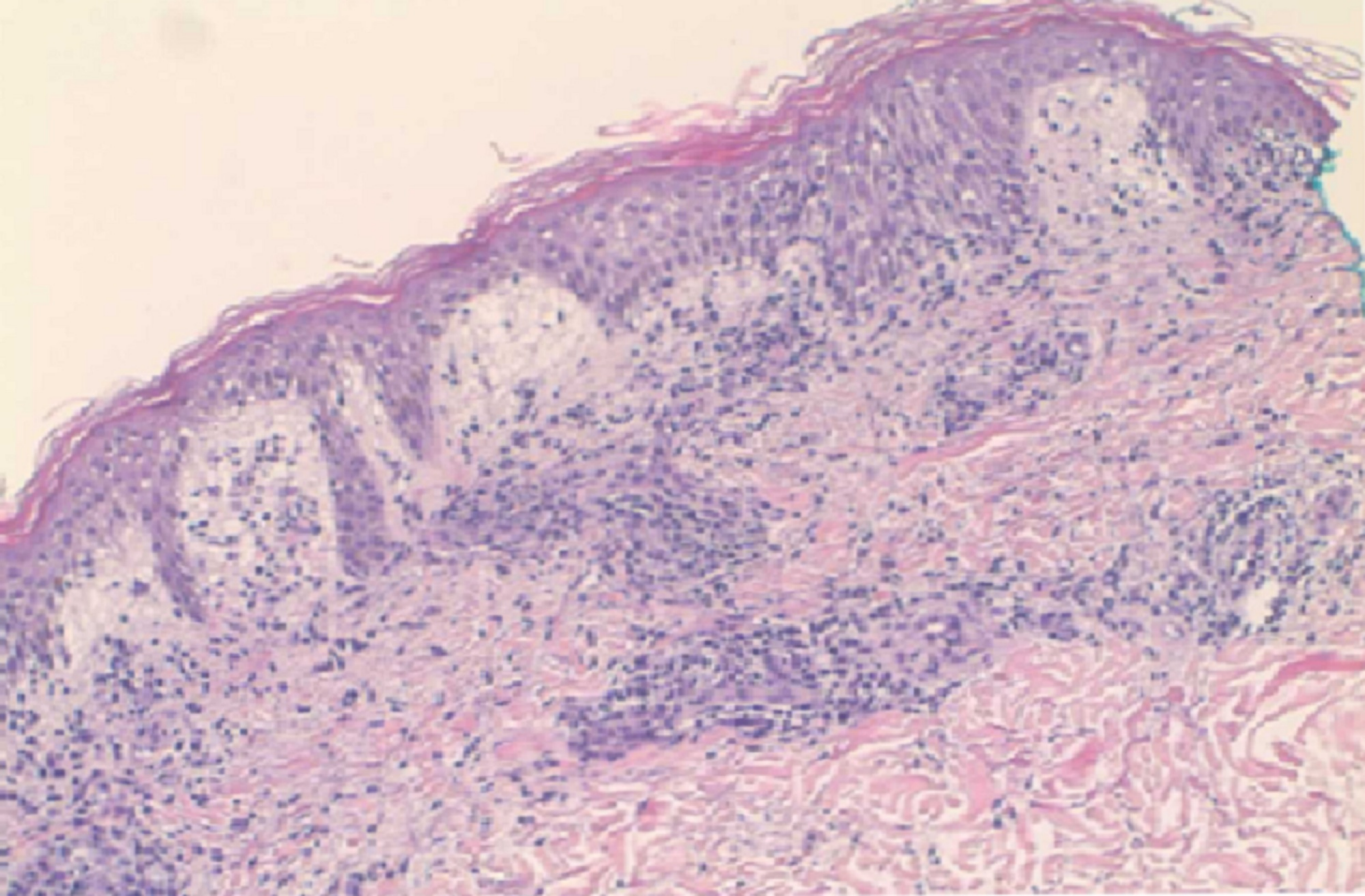
Punch biopsy of the skin in anterior forearm (10x).

A 54-year-old male with a past medical history of dyslipidemia and asthma presented with acute onset of fever, facial fullness, and a generalized rash of two-day duration. He had a recent travel history to Europe (Greece and Turkey), but had no exposure to insects, animals or tick bites. His home medications include rosuvastatin 10 mg daily. His vitals were temperature 102^o^F, pulse 95/min, blood pressure 95/47 mmHg, and respiratory rate 14/min. On physical examination, the patient had scleral icterus, generalized blanching maculopapular rash involving >50% of body surface area, facial fullness, and right upper quadrant tenderness. Laboratory investigations revealed hemoglobin 10 gm/dl, white blood cell count 16.0 K/uL, peripheral eosinophil count 1,700 K/uL, alkaline phosphatase 2,501 U/L, aspartate transaminase 620 U/L, alanine transaminase 680 U/L, total bilirubin 13.2 mg/dl with a direct component of 9 mg/dl, blood urea nitrogen 66 mg/dl, creatinine 5.20 mg/dl, glomerular filtration rate 8 ml/min, and immunoglobulin E (IgE) level 623 IU/mL. Serology for viral hepatitis, Epstein-Barr virus, cytomegalovirus, and human herpesvirus 6 was negative. The patient was started on intravenous normal saline, vancomycin, and piperacillin-tazobactam as he met systemic inflammatory response syndrome criteria, but symptoms did not improve. A computed tomographic scan of the chest, abdomen, and pelvis showed generalized lymphadenopathy (Figure 1). Over the next few days, he deteriorated clinically with worsening renal failure requiring renal replacement therapy. Skin biopsy was performed, antibiotics were discontinued, and the patient was started on intravenous methylprednisolone 120 mg/day and topical clobetasol propionate 0.05% ointment with rapid improvement over several days and was discharged on oral prednisone. Biopsy of skin showed vacuolar and interface dermatitis with perivascular lymphocytic and eosinophilic infiltrate (Figure 2). These findings were consistent with DRESS syndrome. At one-month follow-up, the patient was asymptomatic.

## Discussion

We present a case of DRESS syndrome in a patient with rosuvastatin use. As the onset of fever and rash correlated with recent travel to Europe, infectious etiology was assumed obscuring the correct diagnosis and our initial diagnostic approach. Malignancy and vasculitis became prominent differentials with the emerging evidence of multi-organ dysfunction (liver and kidneys), clinical deterioration, and generalized lymphadenopathy on computer tomography imaging; however, increased eosinophil count and elevated IgE levels were more consistent with DRESS syndrome.

The onset of symptoms in DRESS is usually delayed, ranging from two to six weeks after initiation of the drug [7]. Our patient reported that he was prescribed rosuvastatin a few years ago by his primary physician, but he never took it consistently. He was non-compliant with his rosuvastatin for about one year before starting it again about six weeks before the presentation. One possible explanation could be that inconsistent use of the drug causes initial sensitization and re-exposure causes a severe reaction manifesting as DRESS. It has been shown that following re-exposure, the symptoms may occur more rapidly and maybe more severe [8]. Our patient did not recall having a reaction to rosuvastatin when he first started taking it. He may have had a mild subclinical reaction at that time or the inconsistent use may not have been enough to sensitize his immune system to mount a generalized hypersensitivity reaction as seen in DRESS. According to our literature review, only one case of DRESS associated with atorvastatin use has been reported [9].

RegiSCAR (European Registry of Severe Cutaneous Adverse Reactions) is a scoring system developed to aid in the diagnosis of DRESS (Table 1). It takes into account various clinical features and laboratory investigations and depending on the score, cases of DRESS are grouped into four categories: no case, possible case, probable case, and definite case [2]. In our patient, the calculated RegiSCAR score was >6, indicating a “definite” case of DRESS. The limitation to RegiSCAR criteria is that some of the parameters cannot be assessed until later in the disease course (e.g., skin biopsy findings and disease duration). Hence, it is more useful for retrospectively diagnosing but still serves as a useful tool to guide the collection of clinical data.

**Table 1:** RegiSCAR scoring system for DRESS. (16) Total score <2: excluded; 2-3: possible; 4-5: probable; >6: definite. DRESS, drug reaction with eosinophilia and systemic symptoms; RegiSCAR, European Registry of Severe Cutaneous Adverse Reaction.

Item	Present	Absent
Fever >38.5^o^C	0	-1
Enlarged lymph node ( >1 cm at least two sites)	1	0
Eosinophilia: >700 cells/µL or >10%	1	0
Eosinophilia: >1500 cells/µL or >20%	2	0
Rash >50% body surface area	1	0
>2 of facial edema, purpura, desquamation, or infiltration	1	0
Skin biopsy suggesting alternate diagnosis	-1	0
One organ involvement	1	0
Two or more organ involvement	2	0
Disease duration >15 days	0	-2
Investigation for alternative cause negative	1	0

## Conclusions

Given that statins are not commonly implicated in DRESS syndrome, and the patient’s initial clinical picture seemed to be related to an infectious etiology, there was some delay in diagnosis. Once correctly diagnosed, the patient improved with systemic glucocorticoids and supportive treatment. DRESS syndrome can be associated with significant morbidity and mortality, and hence should be always considered in the differential of cutaneous eruptions with systemic signs and symptoms. It may occur in the setting of drugs not classically known to be associated with it. Hence, a thorough reconciliation of patient’s medication should be performed, and particularly recent medication changes should be explored. The culprit drug should be immediately discontinued, and the reaction should be documented in the health record as a severe allergy to avoid re-exposure. Clinicians should be aware that statins, which are one of the commonest prescription drugs, can potentially be associated with DRESS syndrome.
